# Peste des petits ruminants in Pakistan; past, present and future perspectives

**DOI:** 10.1186/s40781-015-0066-0

**Published:** 2015-11-02

**Authors:** Muhammad Abubakar, Muhammad Irfan, Shumaila Manzoor

**Affiliations:** National Veterinary Laboratories, Park Road, Islamabad, Pakistan; Faculty of Veterinary and Animal Science, PMAS-Arid Agriculture University, Rawalpindi, Pakistan

**Keywords:** PPR, History, Outbreaks, Seroprevalence, Morbidity, Mortality, Diagnosis, Control

## Abstract

Peste des petits ruminants (PPR) is considered to be one of the main constraints to enhancing the productivity of goats and sheep in regions where it is present and becoming endemic. PPR was recognized in Pakistan in early 1990s but got importance during the Participatory Disease Surveillance (PDS) of Rinderpest Eradication Campaign. Lot of research work has been initiated during last decade towards disease epidemiology, risk factor recognition, laboratory diagnosis, vaccination and demonstration of control strategies. Although there are ongoing projects working towards the progressive control of the disease in country yet there is need to have a national level control program for PPR. Also there is need to have comprehensive social economic surveys, disease hot spot recognition and identification of role of other species in disease transmission. With combined efforts of local and national authorities and political will, there is high likelihood that this devastating disease can be controlled and eventually eradicated in near future.

## Introduction

Peste des petits ruminants (PPR) is an acute and highly contagious viral disease of small ruminants such as goats and sheep. PPR virus (PPRV) is a member of the genus Morbillivirus, and family Paramyxoviridae. Similar to other morbilliviruses, PPR virus is capable of destroying entire populations of immunologically immature/innate hosts by causing epidemics that may spoil the economy of a country and weaken both food security and the livelihoods of farmers. Abubakar et al. [3] have reported dramatic consequences with morbidity of 80–90 % and mortality between 50 and 80 % due to infection of PPR virus in small ruminants. In Pakistan, it causes economic losses of Rs 20.5 billion (US$ 0.24 billion) annually. The main transmission routes of PPR virus are oral and aerosol; the oral, nasal and ocular excretions being the key sources of infection [33].

For many years, PPR was considered as an African disease localized mainly in western and central Africa [62]. More recently, it has become endemic across Sub-Saharan Africa, the Middle East, the Arabian Peninsula, Turkey, Iran, Iraq, Pakistan, India, Bangladesh, Tajikistan and Kazakhstan in Central Asia [83]. The presence of PPR virus has been reported in China [87].

PPR, also known as goat plague, is an important disease in Africa [25, 74] and Asia [78], where small ruminants form a considerable portion of livestock population. It mainly affects goats but involvement of sheep is not exceptional. The disease was once thought to be a fairly restricted problem in West Africa, but is now known to exist in most of the West, Central and East Africa, reaching eastwards through Western and South Asia [42]. However, variation in prevalence and severity of PPR outbreaks can be seen due to variations in the sheep and goat husbandry practices within different geographic regions, topography of different areas and other factors.

PPR should be reported to the World Animal Health Organization (OIE) [37]. Due to its rapid spread nature and consequent capacity, it is earlier regarded as List A disease by the Office of International Des Epizooties [70]. In this review, PPR’s history, current status and future perspectives in Pakistan are discussed.

## Review

### History

PPR disease was first noticed in Ivory Coast in West Africa during 2^nd^ World War [46] and was named as pseudo-rinderpest, stomatitis-pneumoenteritis syndrome and pneumoenteritis complex [30]. PPR has been recognized in Pakistan since 1991 when rinderpest like disease in goats was reported in the province of Punjab [24, 71]. This report was based on clinical signs and post mortem findings without laboratory confirmation [18]. Three of these documentations were based on laboratory confirmation [49, 50, 70]; others were based on clinico-epidemiological observations. More recently, other workers have demonstrated the continuing presence of PPRV inflicting substantial economic losses [2, 11, 49, 57, 58, 90, 91].

### Epidemiology

#### Hosts range

Goats and sheep are natural host of PPR virus but goats affected more severely than sheep [61]. Sheep rarely suffer clinical disease [41, 74] although high morbidity and mortality has been reported but exceptionally is assumed that sheep hold innate resistance to clinical disease [77]. Field outbreaks are reported from a zoological collection in Alain [45].

World widely this disease has been reported in Gazelle and deer [13], Antelope and Dorcas Gazelles (Gazella dorcas), Nubian Ibex (Capra ibex nubiana), Laristan sheep (Ovis orientalis laristani), gemsbok (Oryx gazella) and Nigale (Tragelaphinae) [14]. Similarly said disease has been investigated in domesticated/form animals i.e. sheep, goat, cattle buffaloes and camels [6, 53, 57]. Finally evidence of PPR has been found in free living wild animals i.e. Sindh Ibex [8, 70].

### Transmission

Being an acute and highly contagious viral disease of small ruminants, the transmission of PPRV in healthy animals is matter of dire attention. Transmission PPR virus in healthy animals occurred by direct contact with infected animal and contaminated materials i.e. oculo-nasal and oral discharges, the loose faeces, hold large amount of the virus. Small infective droplets release into the air from these secretions and excretions, especially when affected animals cough or sneeze [7, 10, 31, 81]. Likewise, movement of animals play a key role in transmission i.e., purchases, nomads, infected migratory animals etc. Apart from these nutritional deficiencies which lead to poor immunity of animal might be a cause of rapid transmission of PPR virus which results in heavy outbreaks.

### Disease pattern

Movement of animals is determining factor of disease occurrence. In dry season, animals usually travel long distance in search of fodder and water [65]. In humid areas, PPR always occurred in an epizootic form with 80–90 % morbidity and 50–80 % mortality. PPR is often fatal and usually occur as a subclinical in arid and semi arid areas [61]. Young animals between age of three to four months are more susceptible to PPR virus infection [80] due to decrease in natural immunity (maternal anitibodies) [76]. There is constant circulation of virus between ages of 4 to 24 months [82]. High morbidity and mortality has been reported in all of the age groups [13]. Abubakar et al. [5] reported that prevalence of PPR in small ruminants in Pakistan is 40.98 % and disease is severe in goats mostly. Taylor and Abegunde [82] has recorded a prevalence of 57 % in sheep and 44 % in goats during a field survey in Nigeria. Taylor and Barrett [83] have reported that the disease rate of PPR in sheep appears to be more than that in goats. Singh et al. [79] reported an almost similar prevalence for sheep (36.3 %) and goats (32.4 %) in India while Zahur et al. [92] found a higher prevalence in goats (52.9) than in sheep (37.7 %). So in short a regions discrepancy about disease severity is present across the globe and more is linked to regional environment as well as animal breeds.

### Clinical signs

The clinical signs associated with this disease are pyrexia, purulent mucous discharge from the eyes and nose, necrotizing and erosive stomatitis, gastroenteritis, diarrhoea and bronchopneumonia [27].

Clinical examination of affected goats revealed disturbed breathing and cough, muco-purulent discharge from eyes and nose severe diarrhea in young-ones, ulceration on mucous membrane of mouth, fever and depression. The postmortem examination revealed dark red areas (congestion) in different lobes of lungs, small and large intestines [2, 4].

Sometimes PPR misdiagnosed as contagious caprine pleuropneumonia (CCPP), contagious ecthyma or pasteurellosis. This is partially due to lack of awareness, as it is a new disease, but also due to lack of diagnostic tools available to the ordinary district laboratories in Pakistan. PPR virus affects goats severely but mild form of disease in sheep while cause subclinical infection in cattle [20].

### Abortions

Abubakar et al. [2] has reported that serum samples from the aborted dams found positive for PPR antibodies so the PPR disease has a possible association of mortality and prevalence with high rate of abortions in goat. Moreover, if the animal is infected with PPR virus abortions may occur at any stage of gestation.

### Morbidity and mortality

The morbidity rate is 100 % and in severe outbreaks mortality reaches to 100 % [72]. Morbidity and mortality rates vary but may reach up to 100 % [61]. These rates are usually lower in endemic areas (mortality 20 % or less) and sero-surveillance is sometimes the only indicator of the infection [75]. Diallo et al. [38] has reported that in acute cases, mortality varies from 70 to 80 % with death between 10 and 12 days. Moreover the morbidity and mortality rates were higher in sucklers than in adult animals [3].

### Seasonal occurrence

Climatic factors affects PPR occurrence. In rainy season outbreaks minimized due to decreased movement of animals as more fodder availability and increase nutritional and health status. In Dec-Feb the dry and dusty season combine with poor nutrition cause disease spread and cases get peak in April. In Pakistan, Khan et al. [57] reported high PPR seroprevalence in December to February and September and October while Abubakar et al. [5] reported the disease frequency greater in January to April and 33 % of cases reported in March. So we may say that the disease occurrence is throughout the year with the severity variation in different weathers.

### Temporal and spatial distribution of ppr outbreaks

In Pakistan, during the last decade, PPR outbreaks have increased to an alarming level involving newer areas [17]. As, on the basis of clinical and serological methods, an outbreak of PPR was reported in goat flocks of Livestock Production and Research Institute (LPRI), Bahadurnagar, Okara, Pakistan [15] but amazingly no serological evidence of PPR was found in healthy sheep on same form. According to another reports based on observations from 50 laboratory confirmed outbreaks of PPR and provides details of the presence or otherwise of PPR virus (PPRV) in 427 tissue/organ samples from small ruminants in Pakistan. It was concluded that the disease outbreaks were more severe in goats than sheep and the frequency of disease outbreaks was greater between the months of January to April.

Based on the data of 50 outbreaks (427 samples), Abubakar et al. [3] reported the prevalence of PPR in small ruminants in Pakistan was 40.98 %. A greater number of positive cases were observed in the southern and northern parts of the country (30–60 %) as compared to west and south-west (10–30 %).

The OIE World Animal Health in 2000 also confirmed the outbreak of PPR with IcELISA at a wildlife breeding center of Faisalabad, Punjab, Pakistan. Similarly, in district Chitral, North West Frontier Province (NWFP), Pakistan in June 2006, an outbreak of Peste Des Petites Ruminants (PPR) was investigated in goat flocks. Based on competitive and immuno-capture ELISA, 09 (39.15) animal were positive for PPR antibodies [4]. Apart from these detailed reports, details of some significant outbreaks have been mentioned in Table 1.

**Table 1:** Spatial history of PPR outbreaks in various regions of Pakistan

Region/City	Month/Year	Specie affected	Diagnostic test	Reference
Punjab province	1991	Goat	Clinical signs	[24]
Punjab province	1995	Goat	Clinical signs and ELISA	[18]
D.G. Khan	1997	Goat	Clinical signs	[26]
Rawalpindi	1998	Sheep & Goat	Clinical signs and ELISA	[49]
Okara	February, 2005	Goat	cELISA	[15]
Lahore	April, 2006	Goat	AGID	[73]
Islamabad Capital Territory	2006	Sheep	Clinical and ELISA	[91]
Arandu, South West Chitral	2007	Goat	cELISA	[4]
Multan, Faisalabad	2011	Goat	RT-PCR	[64]
Faisalabad, Attock, D.G Khan, Bhakkar, Kasur	Jan-Dec, 2010	Sheep & Goat	RT-PCR and cELISA	[54]
Islamabad Capital Territory	2011	Goat	Clinical, antigen and antibody detection	[9]
Sindh Province	2009−2011	Sheep & Goat	cELISA	[7]
Sindh Province	2009	Sindh Ibex	Clinical, antigen and antibody detection	[8]
Gujranwala	Sep-Dec, 2012	Sheep & Goat	RT-PCR	[16]
Islamabad Capital Territory	2014	Goat	Clinical and antigen detection	[86]
Attock, Texilla, Islamabad Capital Territory	2015	Goat	ELISA and PCR	[12]

### Risk factors of ppr for sheep and goats population

#### Age

Young animals are more affected by the disease and morbidity and mortality rates are much higher. The highest sero-prevalence is usually recorded in animals over 2 years of age. Moreover these older animals are more likely to be seropositive for PPR than young ones [92].

### Specie

Although it is known as the disease affects both species but its prevalence and severity varies among sheep and goats. Prevalence of PPR seen and reported higher in goats as compared to sheep in many studies in various regions of Paksitan [5, 34, 40, 54, 59, 92]. Wild ruminants can also be affected with PPR and a significant outbreak has been documented by Abubakar et al. [8] in which the Sindh Ibex was severely affected with PPR.

### Sex

Jalees et al. [54] has reported that sheep showed higher seropositivity in ewe than in ram. Moreover, this phenomenon has been supported in village based production system in Pakistan by Khan et al. [55] who reported that male are usually slaughtered at early ages and female sheep and goats are retained.

### Season

Although the disease is considered to be endemic in Pakistan yet there are few reports of its seasonal occurrence. In rainy season, PPR incidence decreased due to ample amount of fodder availability lead to increased resistance against disease [5]. Large flock size, animals that visit animal market and inadequate veterinary services are risk factors for PPR disease to occur [92].

### Spatial distribution of ppr in various locations of pakistan

The disease pattern, although both goats and sheep are susceptible to infection and may show disease yet they are not always affected simultaneously, for example, in Africa PPR is seen most commonly in goats, while in western and South Asia sheep are usually the most noticeable victims [42]. But if we see the picture in Pakistan, as somewhere else, PPR affect both goats and sheep but in many villages it is seen that only goats are affected usually [84] and this concept is much supported by findings of Abubakar et al. [2].

In different districts of Sindh province, overall PPR seroprevalence in sheep is 49.5 % as compare to goats which is 56.3 %. According to Obi et al. [68], Durojaiye et al. [39] and Abubakar et al. [7], most cases of PPR emerge with the start of summer season and cases get peak during the months of April to July and then the prevalence drop again.

Khan et al. [57] reported the antibody prevalence of PPR virus in small ruminants in Punjab 51.3 %. The antibodies frequency against PPR virus recorded 67.7 %, 71.1 % and 60.2 % in the months of December, January and February and 50.7 % and 53.0 % in the months of September and October, respectively. Less local fodder availability and poor nutritional status of the animals may play a key role in the transmission of disease. Zahur et al. [92] has reported distribution of PPR virus in different districts of Pakistan that is over all 48.30 %. Jalees et al. [54] investigated that disease is more prevalent in young sheep and goats than adult and predilection site of the PPR virus remained the lymph nodes.

### Sero-prevalence

The true sero-prevalence of PPR in Pakistan estimated to be 48.5 % (95 % CI, 46.6–50.3), and 52.9 % (95 % CI, 50.7–55.1) and 37.7 (95 % CI, 34.4–41.0) for goats and sheep, respectively. The sheep and goats exhibited a different seroprevalence pattern with a quite higher prevalence in goats. The highest prevalence was recorded in animals over 2 years of age: 49.29 % of sheep and 65.94 % of goats were seropositive for PPR [92].

As per report by Abubakar et al. [5] the specie wise PPR antibody seroprevalence recorded in sheep was 54.9 % as compared to goats 44.15 %. The area wise highest seroprevalence was 55.10 % in sheep and goats of Sindh province. The second highest prevalence (76 %) was in Chakwal, followed by 75 %, 64.28 %, 64.71 %, 61.29 %, 60.66 % and 60.31 % in districts Bahawalpur, Haiderabad, Northern areas, Sahiwal, Azad Jammu & Kashmir (AJK) and Rawalpindi, respectively.

According to Zahur et al. [92] PPRV is circulating in the small ruminant population throughout Pakistan. It was found that 49.3 % of sheep and 65.9 % of goats were infected by the third year of their life.

### Comparison of diagnostic options

In general practices, PPR can be diagnosed from its clinical signs, pathological lesions, and specific detection of virus antigen/antibodies/genome in the clinical samples by different serological tests and molecular assays [27]. World widely, diagnostic tests which are used for the detection of PPRV, including isolation on cell culture, agar gel immunodiffusion (AGID), haemagglutination (HA) tests, immunocapture enzyme-linked immunosorbent assay (IC-ELISA), competitive ELISA, virus neutralization test (VNT) and reverse transcriptase polymerase chain reaction (RT-PCR) [19, 44, 67] while in Pakistan, diagnosis of said diseases is done by following methods;

1. Clinical signs and symptoms
2. Post-mortem findings/examinations
3. Laboratory tests: serological, culture and molecular techniques

### Conventional methods and ELISA

At first stage of diagnosis process, following clinical sign and symptom including rapid and labored breathing and cough, muco-purulent discharge from eyes and nose severe diarrhea in young-ones, emaciation, leision in mouth, high fever, lassitude, dyspnoea, anorexia and depression has been varified in various studies/outbreak of PPR in sheep, goat and Sindh ibex in Pakistan. There is possible association of abortions to PPR [2].

The postmortem findings assocated with PPR in Pakistan includes dark red areas and congestin in different lobes of lungs with pnemonetic change, small and large intestines, enlargement of spleen and lymph nodes and erosion of abomasums [2, 15, 56]. A number of serological tests has been practiced in Pakistan for diagnosis of PPR using detecting antigen and antibodies. Among these tests Agar gel immunodiffusion test (AGID), haemagglutination (HA) tests [6, 63], modified Counter immuno-electrophoresis, Immunocapture enzyme linked im-munosorbent assay (IcELISA) [3, 56], Competitive Enzyme Linked Immunosorbent Assay (cELISA) [56, 63, 92], single radial haemolysis test (SRH) and countercurrent immunoelectroosmophoresis (CIEOP), Precipitinogen Inhibition Test (PIT) [63].

Abubakar et al. [5] reported that the competitive ELISA has high diagnostic specificity (99.8 %) and sensitivity (90.5 %) for the detection of PPR virus antibody in convalescent sera when compared with the gold standard VNT [20, 60, 79]. HA test is more sensitive than AGID for detection of PPRV antigen [6]. This result is in concordance with Nussieba et al. [67]. Moreover, the HA test is quick, simple, economical and reliable confirmatory test for the diagnosis of PPR virus.

Munir et al. [63] investigated the comparative efficiency of competitive ELISA (c-ELISA), standard agar gel immunodiffusion (AGID) and precipitinogen inhibition test (PIT) and Aslam et al. [23] investigated comparative efficiency between c-ELISA and AGID for the diagnosis of PPR in Pakistan and concluded that c-ELISA is used as standard since it has the best sensitivity and specificity and can be utilized for samples which are not kept under ideal conditions.

### Serological and molecular diagnostic tools

Serological tests based monitoring; it is difficult to determine the level of vaccine failure and thus aggravates disease epidemiology and its control. As a result, determining the nature of circulating strains in different parts of Pakistan is essential to not only help in disease identification and but also to plan better control strategies in future. For this purpose isolation and molecular characterization of PPR virus is crucial step. Moreover, Genetic characterization of PPRV is very important to understand the epidemiology of PPR outbreaks in Pakistan [16]. These efforts also have been done in Pakistan, as a part of control and eradication of said disease.

As serological tests don’t essentially point out the existing persistence of the disease, it is essential to execute molecular diagnosis along with characterization [21]. That’s way, in recent years, various molecular tools i.e. conventional PCR, real-time PCR [10, 16, 21, 40] has been evaluated for optimal detection of PPR in Pakistan. Genetic analysis of virus proved that lineage IV of PPRV is currently circulating in the country, with certain level of genetic diversity. These Pakistani samples clustered with Chinese, Tajikistani and Iranian isolates [16, 21].

### Genetic characterization

Morbilliviruses are non-segmented, linear, single stranded, −ve sense RNA viruses with genomes 15–16 kb in length and 200 nm in diameter [66]. Similarly PPR virus has a single strand -ve sense RNA genome of ~16 kb (15,948 nucleotides) in length which encodes eight proteins including six contagious, non-overlapping, transcriptional units encoding structural proteins: Nucleoprotein (N), Phosphoprotein (P), Matrix protein (M), Fusion protein (F), Haemagglutinin protein (H) and Large polymerase protein (L) along with two nonstructural proteins C and V [28, 29, 36, 47, 48, 32].

Based on phylogenetic analysis PPR virus has categorized into four lineages as I, II, III and IV. Pakistani PPR virus falls in lineage IV closely related to viruses from regions like Middle East, Arabia, south Asia and China [21, 64, 78, 87, 88].

### Vaccine and vaccination

The PPR considered as endemic diseases of sheep and goat in Pakistan especially in the Punjab province where the population of sheep and goat is higher as compared to other provinces. Different vaccination programs were introduced with the live attenuated Virus belonging to Lineage I. Despite of the strict vaccination programs and other preventive and clinical measures the PPR outbreaks are frequent. Moreover, different type of PPR vaccines including conventional, thermostable, recombinant and edible vaccines has been developed and used from control/eradication of said disease world widely [9, 10, 12, 69].

Currently, vaccination is recommended in certain areas of the country. This vaccination is based on Nig75/1, which belong to lineage II, while field isolates from Pakistan are grouped in lineage IV. Genetic characterization of field strains will provide foundations for construction of vaccines from domestic strains as has recently been practiced in India [21]. An overview of studies based on PPR vaccination has been mentioned below. Rashid et al. (2010) studies the response of locally prepared live attenuated PPR cell culture vaccine in sheep and goats of Pakistan. Vaccine produced high serological titre within 21 days post vaccine and was safe while vaccine titre persisted high for one year post-vaccine. Moreover, [56] reported that PPR vaccination during the face of out break showed significant response to control the problem. The findings are useful towards planning appropriate control of the disease in subsistence farming of small ruminants in NWFP. A homologous vaccine has been developed and tested in field trials. The use of this PPR vaccine is strongly recommended to avoid confusion with Rinderpest during serological survey. It is now commercially available. Furthermore, Asim et al. [22] produced and evaluated a live attenuated cell culture vaccine for providing protection against PPR disease to small ruminants (sheep/goat) which are the species most susceptible to PPR virus. No annoying reactions were observed following vaccination. All vaccinated animals developed high titre of antibodies (PI > 50). So this live attenuated PPR cell culture vaccine can be safely used to immunize small ruminants against PPR disease to minimize the huge economical losses.

While, tissue culture based live freeze-dried PPR virus (*PPR 75–1)* vaccine has been produced by Abbas et al*.* [1] using Vero cell line and checked for validation, safety, sterility and efficacy. They concluded that this PPR vaccine would be an effective tool to limit PPR disease in goats as well as to reduce economic losses due to this disease in Pakistan.

The efficiency of PPRV vaccines available in Pakistan on the basis of the humoral immune response measured by haemagglutination inhibition (HI) and agar gel immunodiffusion (AGID) tests in sheep (*n* = 60) and goats (*n* = 60). Geometric mean titer (GMT) of antibodies against locally manufactured PPRV vaccine was higher (207.9) in comparison with Pestivec (73.3), a vaccine imported from Jordan at 63^rd^ day post vaccination in sheep; the corresponding values in goats were 147.0 and 48.5, respectively. All animals of control group were negative for antibodies by both of the diagnostic tests. Moreover, it was determined that efficacy of PPR virus vaccines depend on proper storage temperature, pH of buffer and immune response is better in sheep than in goats [51].

### Control and prevention

Different control and preventive strategies can be used from PPR in animal. At very first stage separate the infected animals from healthy animal to minimise the chance of transmission of PPR virus from infected animals to healthy animals. Secondly slaughtering of apprent diseases animals and seropositive animals, moreover proper dispose off all infected material and decontamination of items of infected sheep/goat flock is crucial for control/ eradication of PPR. Moreover, vaccination of animals is good option to minimize the risk of occurrence in any healthy animal ppulation. In worldwide different immunization strategies against PPR has been used i.e. earlier immunization of small ruminants was done with lymph node and spleen materials containing virulent virus inactivated with 1.5–5 % chloroform, attenuated tissue culture rinderpest vaccine (TCRV) but now PPR homologous vaccine is available which is prepared by a new freeze-drying process and addition of stabilizing agents [30, 35, 89].

### Economic impact

PPR is considered as one of the major constraints in augmenting the productivity of small ruminants in developing countries and mostly severely affects poor farmer's economy [27, 52]. According to the Food and Agriculture Organization (FAO), 62.5 % of global domestic small ruminant’s population is at risk of being infected by PPR virus. PPR is a target animal disease for poverty alleviation [43]. In one state of India, Maharashtra, annual losses due to PPR were estimated as Rs. 918 and Rs. 945 millions in sheep and goats, respectively [85].

Abubakar and Munir [11] reported the economic loss due to three outbreaks of PPR in Punjab. Disease caused mortality and morbidity of 10–15 % and 20–40 %, respectively, within a time period of 01–03 weeks. At these three farms, 116 of 365 animals exhibited the clinical disease, with an overall morbidity rate of 31.78 %. A total of 43 animals died with mortality rate of 11.78 % (43/365) causing a direct financial loss of $4300 (Pakistan Rupees 430,000/-), while the indirect cost due to treatment, loss of animal body condition, reduction in market value, increase veterinary services and labour was $7911 (Pak Rs. 791,100/-).

### Future perspectives

Currently, although the PPR vaccine production capacity is present in the two places in the country yet there is no organized PPR vaccination campaign going on. With the current population of more than 90 millions of small ruminants and endemic situation of PPR, there is continuous threat from PPR in terms of food security. International authorities working on animal health (OIE and FAO) have recognized PPR as the next target disease for control and possible eradication from the world. So there is need of the time to have national PPR control program in the country. After the successful Rinderpest eradication campaign, OIE has officially declared PPR as next candidate disease, to be eradicated. Therefore serious efforts have been started towards the disease understanding and possible measures for its eradication.

## Conclusion

Although there is a project launched by the FAO for the progressive control of PPR in Pakistan yet there is need to have comprehensive national program to combat this menace. This could only be achieved by the combined efforts of local and national authorities as well as political will; along with continuous support and strengthening by international agencies.
